# On TikTok use disorder tendencies, fear of missing out and everyday cognitive failure

**DOI:** 10.1016/j.abrep.2026.100675

**Published:** 2026-02-07

**Authors:** Yao Wang, Sebastian Markett, Zhiying Zhao, Christian Montag

**Affiliations:** a Centre for Cognitive and Brain Sciences, Institute of Collaborative Innovation, University of Macau, Macau SAR, China; b Department of Psychology, Humboldt-Universität zu Berlin, Berlin, Germany; c Department of Psychology, Faculty of Social Sciences, University of Macau, Macau SAR, China; d Department of Computer and Information Science, Faculty of Science and Technology, University of Macau, Macau SAR, China

**Keywords:** Social media addiction, TikTok, Cognitive failure, Fear of missing out

## Abstract

The present work examines associations between TikTok Use Disorder (TTUD) tendencies, fear of missing out (FoMO) and everyday cognitive failure. In line with prior studies on problematic social media use, we observed in N = 720 TikTok users (249 males, 471 females; mean age = 37.78 years) − who completed the TikTok Use Disorder Questionnaire (TTUD-Q), the FoMO Scale, and the Cognitive Failure Questionnaire (CFQ) − that both higher trait and state FoMO went along with more self-reported cognitive failure in everyday life. This association was mediated by TTUD tendencies, albeit to different degrees. The distinction between general FoMO tendencies and situational, online-related state FoMO appears to be important for understanding putative mechanisms behind the here presented associations and is discussed further. Overall, the findings suggest that higher disordered use tendencies of TikTok may be linked to more cognitive failure, potentially due to frequent app-related interruptions of everyday activities. However, these conclusions are limited by the self-report nature of data and the cross-sectional design which precludes causal inference.

## 1 Introduction

With the rapid rise of social media, an estimated 5 billion people worldwide now use these platforms to communicate, obtain information, and establish social connections (Statista, 2025). The positive role of social media in enhancing social capital and information dissemination efficiency is undeniable (Ellison et al., 2007). However, an increasing number of studies are examining the potentially negative consequences of social media, particularly the possibility that excessive use of social media may lead to symptoms resembling a behavioral addiction (Montag & Markett, 2024). Existing research suggests that differences in platform design may contribute to varying levels of addiction potential across platforms (Montag et al., 2024): TikTok, for example, may be more likely to induce excessive use compared to other platforms due to its short video content and personalized recommendation algorithms (Montag et al., 2021). This excessive use may not only adversely affect users’ mental health but also further impact their daily behavior and cognitive functioning (Wegmann & Brand, 2020). It has been discussed that loss of control and functional impairments might be core criteria to understand addictive like social media use (Moretta & Wegmann, 2025). Against the background of the potential adverse effects of excessive social media use and the rapidly growing TikTok platform (currently more than 1.5 billion users (DataReportal – Global, 2025), it represents a timely endeavor to understand the psychological mechanisms and consequences of excessive use of specific platforms such as TikTok.

### 1.1 The role of fear of missing out in problematic social media/TikTok use

When exploring the mechanisms underlying excessive use of TikTok (and social media platforms more broadly), one key psychological factor is “Fear of Missing Out” (FoMO). FoMO has been defined as the anxiety about missing out on others’ activities or important information (Przybylski et al., 2013). FoMO has both state and trait dimensions (Wegmann et al., 2017). Trait FoMO could be seen as a stable, dispositional tendency to fear missing out while state FoMO, in contrast, could refer to a more situational context-dependent anxiety related specifically to missing out in the online domain. Research indicates that FoMO might significantly increase the frequency of social media use, as users attempt to reduce anxiety through gaining more information about the current state of their social networks on these platforms (Blackwell et al., 2017). For example, social media’s instant feedback mechanisms, such as likes and comments, are associated with repeated logins or extended scrolling as users seek to confirm their social status or access the latest information. Such behavior is associated with increased usage time and may disrupt daily life and cognitive functioning through this psychological factor (Alt, 2015).

### 1.2 Research on TikTok Use Disorder

Previous research has introduced the concept of TikTok Use Disorder (TTUD) tendencies and developed the TikTok Use Disorder Questionnaire (TTUD-Q) which assesses addictive-like behavior on the platform based on the World Health Organization’s (WHO) Gaming Disorder framework (Montag & Markett, 2024). This research contributes to the broader debate on whether the Gaming Disorder criteria can be extended to other forms of problematic online use. Recent research (Montag & Markett, 2023) reported that Social Network Use Disorder (SNUD)^1^ tendencies mediate the relationship between FoMO (trait/state) and cognitive failures (Cognitive Failure Questionnaire, CFQ, which captures lapses in attention and memory; Broadbent et al., 1982). Trait FoMO has been linked to stable personality traits such as neuroticism (Rozgonjuk et al., 2021), potentially fostering addictive-like use, thereby potentially increasing cognitive lapses (Montag & Markett, 2023).

To better understand the association between addictive-like use of TikTok and cognitive failure, it is crucial to consider the behavioral mechanisms linking platform usage to cognitive failures. Specifically, excessive TikTok use may exacerbate cognitive failures not merely through time displacement, but by fostering a habit of frequent smartphone checking. A recent study utilizing objective measures, demonstrated that the frequency of smartphone checking is a more significant predictor of daily cognitive failures than the total duration of use (Hartanto et al., 2023). The fragmented and highly algorithmically curated presentation of TikTok content may reinforce users’ repeated revisiting and brief attentional capture behaviors. This near-continuous state of attentional “foraging” can readily interfere with the maintenance of prolonged focused attention, increase the consumption of cognitive resources, and may thereby be associated with an elevated frequency of everyday cognitive failures.

However, findings regarding these hypothetical mechanisms are primarily based on cross-sectional designs (Montag & Markett, 2023) which limit causal interpretations, and have generally focused on social media in more broader terms, overlooking the specific design features of platforms like TikTok. Longitudinally, a recent study suggests that phone use without internet access for two weeks could already lead to more sustained attention (Castelo et al., 2025). For the present study, we build on the SNUD mediation model which assumes that TTUD tendencies might mediate role the association between FoMO and cognitive failure (Montag & Markett, 2023).

### 1.3 Aim of the present study

In this study we aimed to further clarify the specific role of TTUD tendencies regarding its relationship with FoMO and cognitive failures. We propose a new hypothetical model in which FoMO not only directly influences TTUD tendencies but also indirectly affects cognitive failure (as measured by the CFQ) through its impact on TTUD tendencies. Importantly, we refer here to theoretical causality only, given the cross-sectional nature of our data. More specifically, we hypothesized that FoMO (state/trait) would be positively correlated with TTUD tendencies and the correlation between trait FoMO would be weaker than with state FoMO, as the latter is associated with online FoMO (and TTUD tendencies refers to excessive online behavior). Further, we expected TTUD tendencies to be positively correlated with cognitive failures. Finally, we expected that TTUD tendencies would mediate the relationship between FoMO (state/trait) and CFQ. By introducing TTUD tendencies as a mediator, this study can extend the previous results and contribute to a deeper understanding of the psychological mechanisms of addictive-like TikTok use.

## 2 Methods

The data analyzed in this study were derived from a large-scale survey project, for which we designed a platform to study technology use and cognitive failure. The platform was advertised through various media channels in Germany resulting in a convenience sample for which we cannot rule out self-selection bias (such a bias might affect our data, if only people with certain interests have participated – in our study participants did not receive a monetary incentive, but after the study insights into their CFQ scores compared to others in an anonymous way were provided).

While this project served as the basis for two previous publications (Montag and Markett, 2023, Montag and Markett, 2024) the present analysis addresses a distinct research question with a specific focus on platform-specific mechanisms. Specifically, previous works investigated general Social Networks Use Disorder tendencies in relation to cognitive failure, or TikTok Use Disorder tendencies in relation to personality traits and depression. In contrast, the present study exclusively examines the specific associations between TikTok Use Disorder tendencies, Fear of Missing Out (state/trait), and cognitive failure. This specific constellation of variables and the resulting mediation model have not been analyzed or reported in the prior studies. The study was approved by the local ethics committee at Humboldt University, Berlin, Germany. Research was carried out in line with the Declaration of Helsinki. Participants provided informed e-consent and agreed that their data could be shared anonymously via an open data repository. The current dataset is publicly shared via the Open Science Framework (OSF) platform (see the link at the end of the article). Participation was anonymous, and individuals were informed to participate only if they were 18 years or older.

### 2.1 Participants

The initial dataset including several thousand participants stems from an ongoing data collection. For the present analysis, we applied specific inclusion criteria to focus on participants who use TikTok and completed the FoMO, TTUD-Q, and CFQ measurements. As a first data cleaning step, we included only participants who reported using both social media and TikTok (answered “yes” to both). This resulted in 734 participants. Further exclusions were made as follows: eight participants were excluded due to reporting a third gender, as this group was too small for statistical analysis; one participant was excluded for reporting an implausible age of 432 years; and five participants were excluded for being under 18 years old. After applying these criteria and ensuring complete responses for the relevant questionnaires, the final sample consisted of 720 participants, including 249 males (mean age = 41.55 ± 15.84 years) and 471 females (mean age = 35.79 ± 13.55 years).

### 2.2 Questionnaires

This study used the following questionnaire data, and detailed descriptions and psychometric properties of the questionnaires can be referenced in the original studies (Montag and Markett, 2023, Montag and Markett, 2024).

*Fear of Missing Out Scale (FoMO Scale)*

The FoMO Scale (Wegmann et al., 2017) is used to assess both state and trait aspects of FoMO. The scale includes 12 items, with 5 items measuring trait FoMO and 7 items measuring state FoMO. Participants rate each item on a 5-point Likert scale (1 = strongly disagree to 5 = strongly agree), with higher scores indicating higher levels of FoMO. The German version as used in the cited paper above was provided by Elisa Wegmann (in the present work the following internal consistencies were observed: α = 0.788 and ω = 0.810 for trait FoMO; and α = 0.779 and ω = 0.792 for state FoMO).

*Cognitive Failure Questionnaire (CFQ)*

The Cognitive Failure Questionnaire (CFQ) (Broadbent et al., 1982) is used to measure cognitive lapses in daily life, such as forgetting appointments or being distracted. The German version (Klumb, 1995) consists of 32 items originally rated on a scale from 0 (never) to 4 (very often). In the present data set, responses were logged on a scale from 1 to 5 with higher scores indicating a greater tendency toward cognitive failures. The questionnaire has high internal consistency (α = 0.936, ω = 0.937), with higher scores indicating greater cognitive failure tendencies. We stay with the 1–5 version to make it better comparable with the other scales who also start with 1.

*TikTok Use Disorder Questionnaire (TTUD-Q)*

The TikTok Use Disorder Questionnaire (TTUD-Q) (Montag & Markett, 2024) is based on the WHO’s Gaming Disorder Framework and is used to assess addictive-like behaviors related to TikTok use. The questionnaire consists of 4 items rated on a 5-point Likert scale (1 = never to 5 = very often), primarily measuring symptoms such as loss of control, centering TikTok in life above all other things and functional impairment. Internal consistencies are in the satisfying range (α = 0.829, ω = 0.851), with higher scores indicating a greater tendency toward TTUD tendencies. The German version has been reported in the original work (Montag & Markett, 2024).

### 2.3 Data analysis

All analyses were conducted in Jamovi 2.6.26.0 (https://www.jamovi.org). To examine whether the relationship between FoMO (state/trait) and cognitive failures (CFQ scores) is mediated by TTUD tendencies, we conducted the following analyses.

First, we conducted descriptive analyses for FoMO questionnaire scores (state/trait), TTUD-Q scores, CFQ scores, and age. To assess the distributional properties of these variables, we examined skewness and kurtosis to evaluate normality assumptions. Given the expectation that TTUD-Q scores may exhibit a highly skewed distribution, non-parametric tests were considered appropriate alongside parametric tests to ensure robust findings. To detect differences in FoMO, TTUD tendencies, CFQ scores and age by gender, we conducted independent samples t-tests (parametric) and Mann-Whitney U tests (non-parametric).

Product-moment (parametric) and rank-order (non-parametric) correlations were used to examine the pairwise relationships between FoMO (state/trait), TTUD tendencies, CFQ, and age, providing a preliminary exploration of the associations between variables. All group comparisons and correlation analyses were two-tailed.

For mediation analyses, models were constructed with FoMO (state/trait) score as the independent variable, TTUD-Q as the mediator, and CFQ score as the dependent variable. The models estimated direct effects, indirect effects, and total effects, and calculated 95% confidence intervals. In the main text, only the mediation analysis results without considering the covariates (age and gender) are reported to maintain clarity and focus on the primary relationships, while results including the covariates age and gender in the model are provided in the Supplementary Material (see SF1, SF2, ST2 and ST3).

## 3 Results

Results of descriptive analyses and independent samples *t*-test (see Table 1; Table 2) revealed differences in age, CFQ, trait FoMO, and TTUD-Q scores between males and females. Males had a significantly higher average age than females (M_male = 41.55, SD = 15.84; M_female = 35.79, SD = 13.55, Cohen’s d = 0.40, p < 0.001). On the total CFQ score, females scored significantly higher than males (M_male = 80.39, SD = 19.61; M_female = 89.91, SD = 19.32, Cohen's d = −0.49, p < 0.001), indicating that females reported more cognitive lapses. Females score higher than males in the FoMO trait dimension (M_male = 14.05, SD = 4.37; M_female = 14.87, SD = 4.32, Cohen's d = −0.19, p = 0.017), but not in the FoMO state dimension (Cohen's d = −0.02, p = 0.818). Females also scored higher on TTUD tendencies than males (M_male = 6.37, SD = 3.24; M_female = 6.89, SD = 3.22, Cohen's d = −0.16, p = 0.04). The distributions of all variables were within acceptable ranges for skewness and kurtosis.

**Table 1:** Descriptives statistics for the male and female subsample.

	Gender	N	Missing	Mean	Median	SD	Range	Minimum	Maximum	Skewness	SE	Kurtosis	SE
Age	male	249	0	41.55	42	15.84	63	18	81	0.3589	0.154	−0.6703	0.307
	female	471	0	35.79	33	13.55	67	18	85	0.6888	0.113	−0.0751	0.225
CFQ_Sum	male	249	0	80.39	79	19.61	106	44	150	0.7307	0.154	0.9540	0.307
	female	471	0	89.91	89	19.32	116	43	159	0.4051	0.113	0.1311	0.225
FoMO_Trait	male	249	0	14.05	14	4.37	19	5	24	0.0199	0.154	−0.8161	0.307
	female	471	0	14.87	15	4.32	20	5	25	0.0503	0.113	−0.7133	0.225
FoMO_State	male	249	0	16.34	16	5.21	26	7	33	0.4014	0.154	−0.2591	0.307
	female	471	0	16.44	16	5.56	27	7	34	0.4106	0.113	−0.3754	0.225
TTUD tendencies	male	249	0	6.37	5	3.24	16	4	20	1.7602	0.154	2.9911	0.307
	female	471	0	6.89	6	3.22	16	4	20	1.2361	0.113	1.1403	0.225

**Table 2:** Independent Samples T-Tests/Mann-Whitney U tests investigating gender effects in the variables of interest.

		Statistic	df	p		Effect Size
CFQ_Sum	Student's t	6.258	718	0<.001	Cohen's d	−0.4904
	Mann-Whitney U	41,581		0<.001	Rank biserial correlation	0.29091
FoMO_Trait	Student's t	2.397	718	0.017	Cohen's d	−0.1878
	Mann-Whitney U	52,805		0.028	Rank biserial correlation	0.09951
FoMO_State	Student's t	0.230	718	0.818	Cohen's d	−0.0180
	Mann-Whitney U	58,303		0.899	Rank biserial correlation	0.00574
TTUD tendencies	Student's t	2.055	718	0.040	Cohen's d	−0.1610
	Mann-Whitney U	51,096		0.004	Rank biserial correlation	0.12864
Age	Student's t	5.107^a^	718	0<.001	Cohen's d	0.4001
	Mann-Whitney U	46,451		0<.001	Rank biserial correlation	−0.20785

Note. H_a_ μ_male_ ≠ μ_female_.

^a^Levene's test is significant (p < 0.05), suggesting a violation of the assumption of equal variances.

Correlation analyses demonstrated that trait and state FoMO shared comparable patterns of association with other variables. Specifically, trait FoMO was significantly positively correlated with CFQ (Linear r = 0.328, p < 0.001) and TTUD tendencies (Linear r = 0.383, p < 0.001). Similarly, state FoMO exhibited similar positive (but weaker than the trait) correlations with CFQ (Linear r = 0.176, p < 0.001) and TTUD tendencies (Linear r = 0.290, p < 0.001). Age was negatively correlated with all variables, with the strongest negative correlation observed with TTUD tendencies (Linear r = -0.410, p < 0.001), indicating that older individuals scored lower on these variables. Of note, linear and rank correlations yielded comparable results, therefore only linear correlation coefficients are reported in the text (for both results see Table 3).

**Table 3:** Correlation matrix (correlations controlling for age can be found in the supplement; see ST1).

		CFQ_Sum	FoMO_Trait	FoMO_State	TTUD tendencies	Age
CFQ_Sum	Linear r	—				
	p-value	—				
	Non-linear rho	—				
	p-value	—				
FoMO_Trait	Linear r	0.328***	—			
	p-value	0<.001	—			
	Non-linear rho	0.324***	—			
	p-value	0<.001	—			
FoMO_State	Linear r	0.176***	0.379***	—		
	p-value	0<.001	0<.001	—		
	Non-linear rho	0.154***	0.366***	—		
	p-value	0<.001	0<.001	—		
TTUD tendencies	Linear r	0.299***	0.383***	0.290***	—	
	p-value	0<.001	0<.001	0<.001	—	
	Non-linear rho	0.282***	0.357***	0.251***	—	
	p-value	0<.001	0<.001	0<.001	—	
Age	Linear r	−0.199***	−0.309***	−0.169***	−0.410***	—
	p-value	0<.001	0<.001	0<.001	0<.001	—
	Non-linear rho	−0.185***	−0.305***	−0.192***	−0.467***	—
	p-value	0<.001	0<.001	0<.001	0<.001	—

Note. * p < 0.05, ** p < 0.01, *** p < 0.001.

**Fig. 1:**
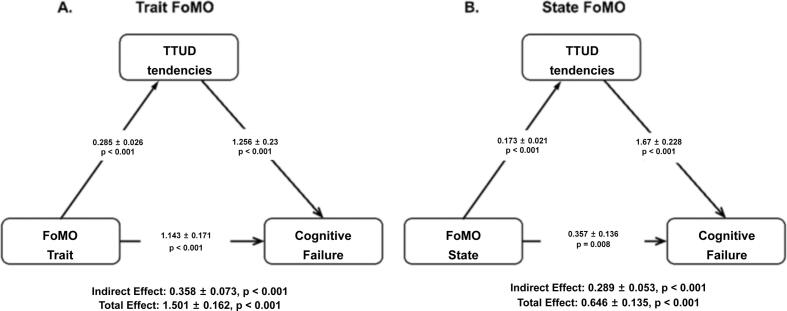
Mediation models for trait FoMO (left side: A) and state FoMO (right side: B). Effect estimates with standard errors are given for the indirect effect, its two components, the direct effect, and the total effect.

Mediation analyses indicated that TTUD tendencies partially mediated the relationship between both state and trait FoMO and CFQ. Specifically, the indirect effect of trait FoMO on CFQ through TTUD tendencies (see Fig. 1A; Table 4) was significant (Estimates = 0.358, SE = 0.073, p < 0.001), and the direct effect was also significant (Estimates = 1.143, SE = 0.171, p < 0.001). Similarly, the indirect effect of state FoMO on CFQ (see Fig. 1B; Table 5) through TTUD tendencies was significant (Estimates = 0.289, SE = 0.053, p < 0.001), with a significant direct effect (Estimates = 0.357, SE = 0.136, p = 0.008). These results support the hypothesis that TTUD tendencies mediate the relationship between trait and state FoMO and cognitive failures. The results from the mediation analyses additionally considering age and gender were consistent with the above results and are detailed in the Supplementary Materials.

**Table 4:** Mediation model with the variables trait FoMO (predictor), TTUD tendencies (mediator) and CFQ (outcome).

				95% C.I. (a)				
Type	Effect	Estimate	SE	Lower	Upper	β	z	p
Indirect	FoMO_Trait ⇒ TTUD ⇒ CFQ_Sum	0.358	0.0730	0.215	0.501	0.0781	4.90	0<.001
Component	FoMO_Trait ⇒ TTUD	0.285	0.0256	0.235	0.335	0.3829	11.12	0<.001
	TTUD ⇒ CFQ_Sum	1.256	0.2301	0.805	1.707	0.2039	5.46	0<.001
Direct	FoMO_Trait ⇒ CFQ_Sum	1.143	0.1712	0.808	1.479	0.2495	6.68	0<.001
Total	FoMO_Trait ⇒ CFQ_Sum	1.501	0.1615	1.185	1.818	0.3276	9.30	0<.001

Note. Confidence intervals computed with method: Standard (Delta method).

Note. Betas are completely standardized effect sizes.

**Table 5:** Mediation model with the variables state FoMO (predictor), TTUD tendencies (mediator) and CFQ (outcome).

				95% C.I. (a)				
Type	Effect	Estimate	SE	Lower	Upper	β	z	p
Indirect	FoMO_State⇒ TTUD ⇒ CFQ_Sum	0.289	0.0530	0.1849	0.393	0.0787	5.45	0<.001
Component	FoMO_State ⇒ TTUD	0.173	0.0212	0.1312	0.214	0.2904	8.14	0<.001
	TTUD ⇒ CFQ_Sum	1.670	0.2278	1.2238	2.117	0.2712	7.33	0<.001
Direct	FoMO_State ⇒ CFQ_Sum	0.357	0.1356	0.0911	0.623	0.0973	2.63	0.008
Total	FoMO_State ⇒ CFQ_Sum	0.646	0.1346	0.3817	0.909	0.1761	4.80	0<.001

Note. Confidence intervals computed with method: Standard (Delta method).

Note. Betas are completely standardized effect sizes.

## 4 Discussion

The goal of this study was to explore how TTUD tendencies mediated the relationship between FoMO (state/trait) and CFQ. We found that both trait and state FoMO were significantly positively correlated with TTUD tendencies, and TTUD tendencies were also positively correlated with cognitive failures. These results align with previous research on excessive general social media use (Hussain et al., 2020, Montag and Markett, 2023). In line with this, the mediation analyses in this study showed that TTUD tendencies partially mediate the relationship between FoMO (state/trait) and CFQ, supporting the idea of a FoMO–excessive TikTok use–cognitive failure pathway. This pathway suggests that anxiety related to FoMO may be linked to reduced cognitive resources due to excessive TikTok use, whereas the latter is likely associated with distractions, memory errors, and other everyday cognitive failures. Specifically, trait FoMO may be associated with long-term TikTok use through ongoing fear of missing out in many areas of one’s own life (perhaps also with carry over effects from the analogue to the online world), engaging attentional resources and relating to more cognitive failures (Montag & Markett, 2023). Meanwhile, state FoMO may be linked to immediate online anxiety leading to frequent TikTok checks, creating short-term disruptions in attention (Wegmann et al., 2017). This mediation mechanism is consistent with the I-PACE (Person-Affect-Cognition-Execution) model, emphasizing that emotional motives (e.g., FoMO) are related to functional impairment through behavioral dyscontrol (TTUD tendencies) (Brand et al., 2016). This finding builds on the observations of Montag and Markett about SNUD mediating the FoMO and cognitive failures association, applying it to the TikTok-specific context and highlighting the importance of platform-specific research (Montag & Markett, 2023).

Notably, our study found that the correlation between TTUD tendencies and trait FoMO was stronger than its correlation with state FoMO. This pattern contrasts with findings by Montag and Markett (Montag & Markett, 2023), where tendencies for general SNUD, measured by the SNS Addiction Test (SNS-AT), showed a stronger correlation with state FoMO than with trait FoMO. This previous study suggested that state FoMO reflects immediate anxiety related to online environments, such as the need for connection triggered by real-time notifications, which aligns with the design of general social media platforms (Wegmann et al., 2017). To better understand these seemingly conflicting findings, we took advantage of the fact that our data set also included the SNS-AT, a measure of general social media overuse as employed in our earlier work. In post-hoc analyses (see Supplement Table 5), we examined correlations between the SNS-AT and both trait and state FoMO. Replicating earlier findings, we observed that trait FoMO is less associated with SNUD (SNS-AT) than state FoMO (but in the present work only a small difference on correlational sum level analysis could be observed; compare to Montag & Markett, 2023). Interestingly, for TTUD tendencies we found the reverse pattern: trait FoMO showed a stronger association than state FoMO. A deeper analysis of items of the SNS-AT revealed that among the six items of the SNS-AT (salience, tolerance, mood modification, relapse, withdrawal, conflict), salience, tolerance and withdrawal showed stronger correlations with state FoMO than with trait FoMO. This suggests that SNS-AT (in parts) may capture immediate emotional responses and behavioral urges tied to state FoMO. In contrast, all four items of the TTUD-Q (loss of control, priority over other things, upholding negative behavior despite negative consequences, functional impairment) demonstrated stronger correlations with trait FoMO than with state FoMO (see Supplementary Table 4). Notably, within the correlations with trait FoMO, TTUD-Q items 1 (loss of control) and 2 (priority over other things) exhibited stronger associations than TTUD-Q items 3 (keep on with TikTok use despite problems) and 4 (severe consequences). These findings indicate that the TTUD-Q may be particularly sensitive to trait FoMO, which align with the observed associations between high neuroticism/low conscientiousness and higher trait FoMO (indicating that FoMO is linked to personality; Rozgonjuk et al., 2021) and observations that personality plays a role for understanding TTUD (Montag & Markett, 2024). This differential pattern suggests that the reverse correlation observed with TTUD tendencies might be primarily due to the specific symptoms captured by the TTUD-Q (being inspired by the Gaming Disorder framework by the WHO). This needs to be seen in light of observations that some symptoms of the SNS-AT and of the TTUD tendencies are conceptually closely linked to each other (correlation analyses reported in the Supplement Table 6; item 1 of the TTUD tendencies and item 4 of the SNS-AT assessing loss of control/relapse and item 4 of the TTUD tendencies and item 6 of the SNS-AT tapping into problems in everyday life due to social media use), but these items show opposite associations with trait/state FoMO: While item 1 of TTUD-Q correlates more with trait FoMO than state, the difference between SNS-AT item 4's correlations with FoMO trait and FoMO state is less pronounced. This suggests TikTok’s design may amplify trait FoMO’s role, while SNS-AT’s unique dimensions like withdrawal emphasize state FoMO’s influence. It might be the platform TikTok vs. social media in general character being responsible for the different findings. But it could also be the symptoms being assessed in the SNS-AT, but not in the TTUD tendencies (e.g. mood modification or tolerance).

The present cross-sectional design does not allow us to infer the directional relationships between these variables, or alternative models, such as CFQ resulting in more FoMO, would also be plausible. For instance, previous work (Hadlington, 2015) found that individuals with high levels of cognitive failures in daily life might be more susceptible to problematic mobile phone use, suggesting that pre-existing deficits in attention and self-regulation may act as antecedents to addiction (but see also cross-sectional data here). This possibility is further supported by recent longitudinal evidence demonstrating a bidirectional relationship between problematic social media use and cognitive failures (Kasturiratna & Hartanto, 2025). Similarly, excessive engagement with social media could reinforce maladaptive psychological states through constant social comparison mechanisms (Vogel et al., 2014), potentially intensifying FoMO over time. However, our proposed directionality is grounded in the I-PACE model (Brand et al., 2019), which posits that predisposing variables—such as personality traits and psychopathology—act as core determinants that trigger specific internet-use behaviors (and we argue for FoMO being part of personality; Rozgonjuk et al., 2021). We acknowledge that alternative models with different orderings of variables could be tested and might be meaningful. To support such future work, the data is made publicly available, allowing interested researchers to explore additional model specifications. As outlined in the introduction, we consider the variable ordering used in the present analysis to be the most theoretically grounded. A further limitation of the study lies in its exclusive reliance on self-report measures, which may be influenced by limited introspective accuracy or tendencies toward desirable responding.

In conclusion, our findings extend previous observations showing that tendencies toward SNUD mediate the relationship between FoMO (trait and state) and cognitive failure to the specific case of TikTok overuse. This distinction is important, as general questionnaires on social media addiction may lead participants to consider a wide range of platforms, potentially conflating distinct usage patterns. Platform-specific assessments, such as the one used here, allow for a more precise understanding of these dynamics. Given the ongoing regulatory scrutiny of TikTok’s potentially addictive design, we believe that the focus and timing of this study is highly relevant.

## CRediT authorship contribution statement

**Yao Wang:** Writing – review & editing, Writing – original draft, Visualization, Formal analysis. **Sebastian Markett:** Writing – review & editing, Validation. **Zhiying Zhao:** Writing – review & editing, Validation. **Christian Montag:** Writing – review & editing, Validation, Supervision, Resources, Project administration, Methodology, Investigation, Funding acquisition, Data curation, Conceptualization.

## Funding

The present work is supported by grant SRG2025-00018-ICI by University of Macau.

## Declaration of competing interest

Dr. Montag reports no conflict of interest. However, for reasons of transparency Dr. Montag mentions that he has received (earlier to Ulm University and University of Bonn) grants from agencies such as the German Research Foundation (DFG). Dr. Montag has performed grant reviews for several agencies; has edited journal sections and articles; has given academic lectures in clinical or scientific venues or companies; and has generated books or book chapters for publishers of mental health texts. For some of these activities he received royalties, but never from gaming or social media companies. Dr. Montag mentions that he was part of a discussion circle (Digitalität und Verantwortung: https://about.fb.com/de/news/h/gespraechskreis-digitalitaet-und-verantwortung/) debating ethical questions linked to social media, digitalization and society/democracy at Facebook. In this context, he received no salary for his activities.

## Data Availability

Data is available at the Open Science Framework: https://osf.io/yr8qm/.

## Footnotes

1 A term proposed for social media addiction.
