# Supplementary material for: On TikTok use disorder tendencies, fear of missing out and everyday cognitive failure
Source: Addict Behav Rep. 2026 Feb 7;23:100675. doi: 10.1016/j.abrep.2026.100675 (PMC12991948; doi:10.1016/j.abrep.2026.100675)
Supplement: Supplementary Data 1 [file mmc1.docx]

| Supplementary Table 1 Partial Correlation for controlling variable ‘Age’ | | | | | |
| --- | --- | --- | --- | --- | --- |
|  |  | **CFQ_Sum** | **FoMO_Trait** | **FoMO_State** | **TTUD** |
| **CFQ_Sum** | **Pearson's r** | — |  |  |  |
|  | **p-value** | — |  |  |  |
|  | **Spearman's rho** | — |  |  |  |
|  | **p-value** | — |  |  |  |
| **FoMO_Trait** | **Pearson's r** | 0.285*** | — |  |  |
|  | **p-value** | <.001 | — |  |  |
|  | **Spearman's rho** | 0.286*** | — |  |  |
|  | **p-value** | <.001 | — |  |  |
| **FoMO_State** | **Pearson's r** | 0.148*** | 0.348*** | — |  |
|  | **p-value** | <.001 | <.001 | — |  |
|  | **Spearman's rho** | 0.123*** | 0.329*** | — |  |
|  | **p-value** | <.001 | <.001 | — |  |
| **TTUD** | **Pearson's r** | 0.244*** | 0.295*** | 0.246*** | — |
|  | **p-value** | <.001 | <.001 | <.001 | — |
|  | **Spearman's rho** | 0.226*** | 0.255*** | 0.186*** | — |
|  | **p-value** | <.001 | <.001 | <.001 | — |
| Note. controlling for 'Age' | | | | | |
| Note. * p < .05, ** p < .01, *** p < .001 | | | | | |

Supplementary Figure 1 Conceptual Diagram of FoMO_Trait ⇒ TTUD ⇒ CFQ_Sum


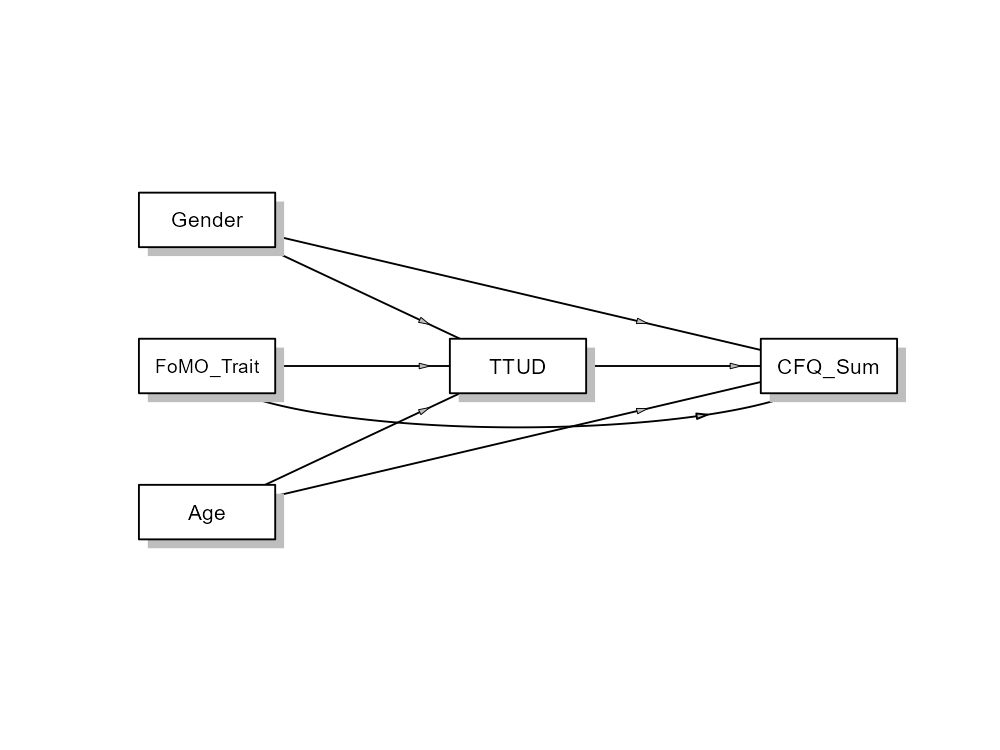


| Supplementary Table 2 Generalized linear model mediation analysis with the variables trait FoMO (independent variable), TTUD (mediator), CFQ (dependent variable), age (covariates), gender (factor) | | | | | | | | |
| --- | --- | --- | --- | --- | --- | --- | --- | --- |
|  | | | | **95% C.I. (a)** | |  | | |
| **Type** | **Effect** | **Estimate** | **SE** | **Lower** | **Upper** | **β** | **z** | **p** |
| **Indirect** | **Gender ⇒ TTUD ⇒ CFQ_Sum** | -0.0751 | 0.26336 | -0.5912 | 0.44113 | -0.00179 | -0.285 | 0.776 |
|  | **FoMO_Trait ⇒ TTUD ⇒ CFQ_Sum** | 0.2466 | 0.05845 | 0.1320 | 0.36114 | 0.05381 | 4.219 | <.001 |
|  | **Age ⇒ TTUD ⇒ CFQ_Sum** | -0.0838 | 0.01931 | -0.1216 | -0.04595 | -0.06151 | -4.340 | <.001 |
| **Component** | **Gender ⇒ TTUD** | -0.0642 | 0.22487 | -0.5049 | 0.37654 | -0.00944 | -0.285 | 0.775 |
|  | **TTUD ⇒ CFQ_Sum** | 1.1692 | 0.23865 | 0.7015 | 1.63697 | 0.18983 | 4.899 | <.001 |
|  | **FoMO_Trait ⇒ TTUD** | 0.2109 | 0.02542 | 0.1611 | 0.26072 | 0.28348 | 8.296 | <.001 |
|  | **Age ⇒ TTUD** | -0.0717 | 0.00766 | -0.0867 | -0.05665 | -0.32405 | -9.353 | <.001 |
| **Direct** | **Gender ⇒ CFQ_Sum** | 7.9364 | 1.44005 | 5.1140 | 10.75885 | 0.18954 | 5.511 | <.001 |
|  | **FoMO_Trait ⇒ CFQ_Sum** | 1.0713 | 0.17038 | 0.7374 | 1.40529 | 0.23380 | 6.288 | <.001 |
|  | **Age ⇒ CFQ_Sum** | -0.0186 | 0.05196 | -0.1204 | 0.08325 | -0.01365 | -0.358 | 0.720 |
| **Total** | **Gender ⇒ CFQ_Sum** | 7.8614 | 1.46479 | 4.9904 | 10.73229 | 0.18775 | 5.367 | <.001 |
|  | **FoMO_Trait ⇒ CFQ_Sum** | 1.3179 | 0.16559 | 0.9934 | 1.64247 | 0.28761 | 7.959 | <.001 |
|  | **Age ⇒ CFQ_Sum** | -0.1024 | 0.04991 | -0.2002 | -0.00457 | -0.07516 | -2.051 | 0.040 |
|  | | | | | | | | |

Supplementary Figure 2 Conceptual Diagram of FoMO_State ⇒ TTUD ⇒ CFQ_Sum


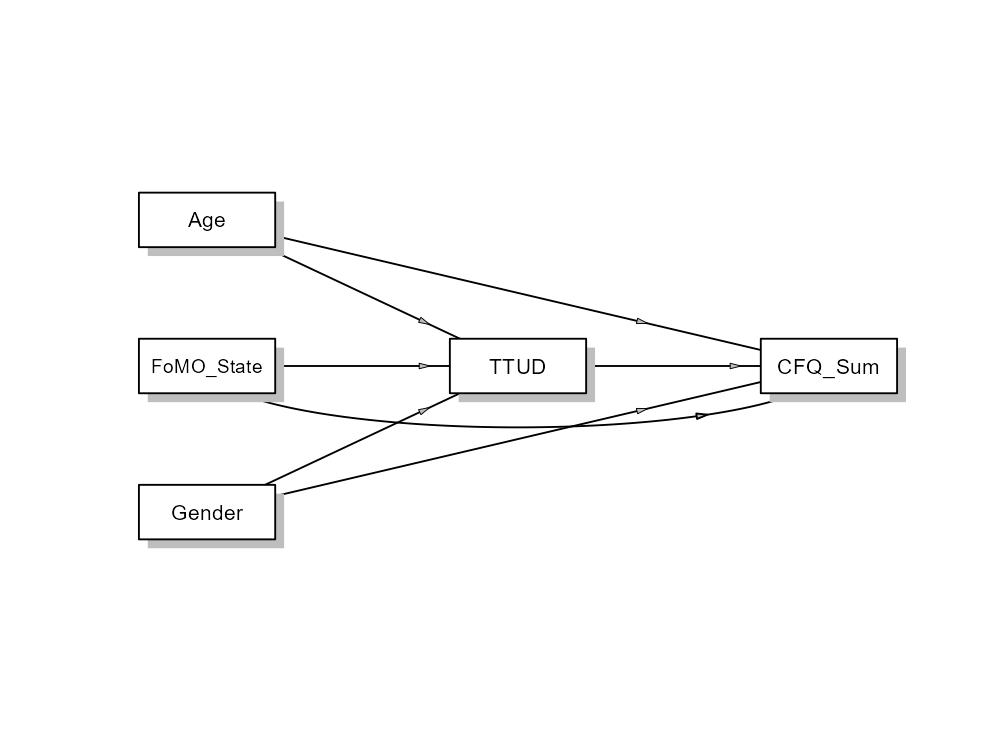


| Supplementary Table 3 Generalized linear model mediation analysis with the variables state FoMO (independent variable), TTUD (mediator), CFQ (dependent variable), age (covariates), gender (factor) | | | | | | | | |
| --- | --- | --- | --- | --- | --- | --- | --- | --- |
|  | | | | **95% C.I. (a)** | |  | | |
| **Type** | **Effect** | **Estimate** | **SE** | **Lower** | **Upper** | **β** | **z** | **p** |
| **Indirect** | **Age ⇒ TTUD ⇒ CFQ_Sum** | -0.1190 | 0.02253 | -0.1632 | -0.0749 | -0.08739 | -5.283 | <.001 |
|  | **FoMO_State ⇒ TTUD ⇒ CFQ_Sum** | 0.1970 | 0.04357 | 0.1116 | 0.2824 | 0.05374 | 4.522 | <.001 |
|  | **Gender ⇒ TTUD ⇒ CFQ_Sum** | 0.0508 | 0.33136 | -0.5987 | 0.7002 | 0.00121 | 0.153 | 0.878 |
| **Component** | **Age ⇒ TTUD** | -0.0820 | 0.00753 | -0.0967 | -0.0672 | -0.37059 | -10.889 | <.001 |
|  | **TTUD ⇒ CFQ_Sum** | 1.4525 | 0.24040 | 0.9813 | 1.9236 | 0.23581 | 6.042 | <.001 |
|  | **FoMO_State ⇒ TTUD** | 0.1356 | 0.01990 | 0.0966 | 0.1746 | 0.22791 | 6.817 | <.001 |
|  | **Gender ⇒ TTUD** | 0.0349 | 0.22806 | -0.4120 | 0.4819 | 0.00514 | 0.153 | 0.878 |
| **Direct** | **Age ⇒ CFQ_Sum** | -0.0665 | 0.05240 | -0.1692 | 0.0362 | -0.04880 | -1.269 | 0.205 |
|  | **FoMO_State ⇒ CFQ_Sum** | 0.3580 | 0.13244 | 0.0985 | 0.6176 | 0.09766 | 2.703 | 0.007 |
|  | **Gender ⇒ CFQ_Sum** | 8.3506 | 1.47114 | 5.4672 | 11.2340 | 0.19943 | 5.676 | <.001 |
| **Total** | **Age ⇒ CFQ_Sum** | -0.1855 | 0.04980 | -0.2831 | -0.0879 | -0.13619 | -3.725 | <.001 |
|  | **FoMO_State ⇒ CFQ_Sum** | 0.5551 | 0.13166 | 0.2970 | 0.8131 | 0.15141 | 4.216 | <.001 |
|  | **Gender ⇒ CFQ_Sum** | 8.4014 | 1.50900 | 5.4438 | 11.3589 | 0.20064 | 5.568 | <.001 |

| Supplementary Table 4 Item level correlation analyses for TTUD-Q and FoMO (trait/state) | | | | | | | |
| --- | --- | --- | --- | --- | --- | --- | --- |
|  |  | **TTUD-Q1** | **TTUD-Q2** | **TTUD-Q3** | **TTUD-Q4** | **FoMO_Trait** | **FoMO_State** |
| **TTUD-Q1** | **Spearman's rho** | — |  |  |  |  |  |
|  | **p-value** | — |  |  |  |  |  |
| **TTUD-Q2** | **Spearman's rho** | 0.733*** | — |  |  |  |  |
|  | **p-value** | <.001 | — |  |  |  |  |
| **TTUD-Q3** | **Spearman's rho** | 0.556*** | 0.601*** | — |  |  |  |
|  | **p-value** | <.001 | <.001 | — |  |  |  |
| **TTUD-Q4** | **Spearman's rho** | 0.394*** | 0.466*** | 0.552*** | — |  |  |
|  | **p-value** | <.001 | <.001 | <.001 | — |  |  |
| **FoMO_Trait** | **Spearman's rho** | 0.315*** | 0.357*** | 0.284*** | 0.251*** | — |  |
|  | **p-value** | <.001 | <.001 | <.001 | <.001 | — |  |
| **FoMO_State** | **Spearman's rho** | 0.219*** | 0.264*** | 0.249*** | 0.196*** | 0.366*** | — |
|  | **p-value** | <.001 | <.001 | <.001 | <.001 | <.001 | — |
| Note. * p < .05, ** p < .01, *** p < .001 | | | | | | | |

| Supplementary Table 5 Correlation analyses for SNS-AT and FoMO (trait/state) | | | | | | | | | | | |
| --- | --- | --- | --- | --- | --- | --- | --- | --- | --- | --- | --- |
|  | |  | **SNS-AT1** | **SNS-AT2** | **SNS-AT3** | **SNS-AT4** | **SNS-AT5** | **SNS-AT6** | **SNS-AT_Sum** | **FoMO_Trait** | **FoMO_State** |
| **SNS-AT1** | | **Spearman's rho** | — |  |  |  |  |  |  |  |  |
|  |  | **p-value** | — |  |  |  |  |  |  |  |  |
| **SNS-AT2** | | **Spearman's rho** | 0.450*** | — |  |  |  |  |  |  |  |
|  |  | **p-value** | <.001 | — |  |  |  |  |  |  |  |
| **SNS-AT3** | | **Spearman's rho** | 0.423*** | 0.660*** | — |  |  |  |  |  |  |
|  |  | **p-value** | <.001 | <.001 | — |  |  |  |  |  |  |
| **SNS-AT4** | | **Spearman's rho** | 0.392*** | 0.447*** | 0.445*** | — |  |  |  |  |  |
|  |  | **p-value** | <.001 | <.001 | <.001 | — |  |  |  |  |  |
| **SNS-AT5** | | **Spearman's rho** | 0.449*** | 0.474*** | 0.450*** | 0.473*** | — |  |  |  |  |
|  |  | **p-value** | <.001 | <.001 | <.001 | <.001 | — |  |  |  |  |
| **SNS-AT6** | | **Spearman's rho** | 0.367*** | 0.392*** | 0.364*** | 0.448*** | 0.394*** | — |  |  |  |
|  |  | **p-value** | <.001 | <.001 | <.001 | <.001 | <.001 | — |  |  |  |
| **SNS-AT_Sum** | | **Spearman's rho** | 0.694*** | 0.784*** | 0.793*** | 0.735*** | 0.714*** | 0.613*** | — |  |  |
|  |  | **p-value** | <.001 | <.001 | <.001 | <.001 | <.001 | <.001 | — |  |  |
| **FoMO_Trait** | | **Spearman's rho** | 0.349*** | 0.360*** | 0.391*** | 0.351*** | 0.332*** | 0.285*** | 0.475*** | — |  |
|  |  | **p-value** | <.001 | <.001 | <.001 | <.001 | <.001 | <.001 | <.001 | — |  |
| **FoMO_State** | | **Spearman's rho** | 0.430*** | 0.372*** | 0.341*** | 0.312*** | 0.447*** | 0.257*** | 0.487*** | 0.366*** | — |
|  |  | **p-value** | <.001 | <.001 | <.001 | <.001 | <.001 | <.001 | <.001 | <.001 | — |
| Note. * p < .05, ** p < .01, *** p < .001 | | | | | | | | | | | |

| Supplementary Table 6 Correlation analyses for TTUD-Q and SNS-AT | | | | | | | | | | | | | |
| --- | --- | --- | --- | --- | --- | --- | --- | --- | --- | --- | --- | --- | --- |
|  |  | **TTUD-Q1** | **TTUD-Q2** | **TTUD-Q3** | **TTUD-Q4** | **SNS-AT1** | **SNS-AT2** | **SNS-AT3** | **SNS-AT4** | **SNS-AT5** | **SNS-AT6** | **TTUD_Sum** | **SNS-AT_Sum** |
| **TTUD-Q1** | **Spearman's rho** | — |  |  |  |  |  |  |  |  |  |  |  |
|  | **p-value** | — |  |  |  |  |  |  |  |  |  |  |  |
| **TTUD-Q2** | **Spearman's rho** | 0.733*** | — |  |  |  |  |  |  |  |  |  |  |
|  | **p-value** | <.001 | — |  |  |  |  |  |  |  |  |  |  |
| **TTUD-Q3** | **Spearman's rho** | 0.556*** | 0.601*** | — |  |  |  |  |  |  |  |  |  |
|  | **p-value** | <.001 | <.001 | — |  |  |  |  |  |  |  |  |  |
| **TTUD-Q4** | **Spearman's rho** | 0.394*** | 0.466*** | 0.552*** | — |  |  |  |  |  |  |  |  |
|  | **p-value** | <.001 | <.001 | <.001 | — |  |  |  |  |  |  |  |  |
| **SNS-AT1** | **Spearman's rho** | 0.241*** | 0.244*** | 0.258*** | 0.213*** | — |  |  |  |  |  |  |  |
|  | **p-value** | <.001 | <.001 | <.001 | <.001 | — |  |  |  |  |  |  |  |
| **SNS-AT2** | **Spearman's rho** | 0.292*** | 0.305*** | 0.254*** | 0.223*** | 0.450*** | — |  |  |  |  |  |  |
|  | **p-value** | <.001 | <.001 | <.001 | <.001 | <.001 | — |  |  |  |  |  |  |
| **SNS-AT3** | **Spearman's rho** | 0.421*** | 0.394*** | 0.320*** | 0.250*** | 0.423*** | 0.660*** | — |  |  |  |  |  |
|  | **p-value** | <.001 | <.001 | <.001 | <.001 | <.001 | <.001 | — |  |  |  |  |  |
| **SNS-AT4** | **Spearman's rho** | 0.414*** | 0.388*** | 0.351*** | 0.259*** | 0.392*** | 0.447*** | 0.445*** | — |  |  |  |  |
|  | **p-value** | <.001 | <.001 | <.001 | <.001 | <.001 | <.001 | <.001 | — |  |  |  |  |
| **SNS-AT5** | **Spearman's rho** | 0.256*** | 0.294*** | 0.246*** | 0.194*** | 0.449*** | 0.474*** | 0.450*** | 0.473*** | — |  |  |  |
|  | **p-value** | <.001 | <.001 | <.001 | <.001 | <.001 | <.001 | <.001 | <.001 | — |  |  |  |
| **SNS-AT6** | **Spearman's rho** | 0.298*** | 0.309*** | 0.380*** | 0.365*** | 0.367*** | 0.392*** | 0.364*** | 0.448*** | 0.394*** | — |  |  |
|  | **p-value** | <.001 | <.001 | <.001 | <.001 | <.001 | <.001 | <.001 | <.001 | <.001 | — |  |  |
| **TTUD_Sum** | **Spearman's rho** | 0.933*** | 0.868*** | 0.704*** | 0.521*** | 0.269*** | 0.334*** | 0.451*** | 0.436*** | 0.297*** | 0.355*** | — |  |
|  | **p-value** | <.001 | <.001 | <.001 | <.001 | <.001 | <.001 | <.001 | <.001 | <.001 | <.001 | — |  |
| **SNS-AT_Sum** | **Spearman's rho** | 0.445*** | 0.442*** | 0.397*** | 0.318*** | 0.694*** | 0.784*** | 0.793*** | 0.735*** | 0.714*** | 0.613*** | 0.490*** | — |
|  | **p-value** | <.001 | <.001 | <.001 | <.001 | <.001 | <.001 | <.001 | <.001 | <.001 | <.001 | <.001 | — |
| Note. * p < .05, ** p < .01, *** p < .001 | | | | | | | | | | | | | |
